# Climatic‐niche evolution with key morphological innovations across clades within *Scutiger boulengeri* (Anura: Megophryidae)

**DOI:** 10.1002/ece3.7838

**Published:** 2021-07-01

**Authors:** Xiuqin Lin, Chungkun Shih, Yinmeng Hou, Xiaoxiao Shu, Meihua Zhang, Junhua Hu, Jianping Jiang, Feng Xie

**Affiliations:** ^1^ CAS Key Laboratory of Mountain Ecological Restoration and Bioresource Utilization and Ecological Restoration Biodiversity Conservation Key Laboratory of Sichuan Province Chengdu Institute of Biology Chinese Academy of Sciences Chengdu China; ^2^ University of Chinese Academy of Sciences Beijing China; ^3^ College of Life Sciences and Academy for Multidisciplinary Studies Capital Normal University Beijing China; ^4^ Department of Paleobiology National Museum of Natural History Smithsonian Institution Washington DC USA; ^5^ Mangkang Ecological Station Tibet Ecological Safety Monitor Network Changdu China

**Keywords:** intraspecific niche evolution, niche expansion, niche shifts, phenotypic plasticity, trait evolution

## Abstract

The studies of climatic‐niche shifts over evolutionary time accompanied by key morphological innovations have attracted the interest of many researchers recently. We applied ecological niche models (ENMs), ordination method (environment principal component analyses; PCA‐env), combined phylogenetic comparative methods (PCMs), and phylogenetic generalized least squares (PGLS) regression methods to analyze the realized niche dynamics and correspondingly key morphological innovations across clades within *Scutiger boulengeri* throughout their distributions in Qinghai–Tibet Plateau (QTP) margins of China. Our results show there are six clades in *S. boulengeri* and obvious niche divergences caused by niche expansion in three clades. Moreover, in our system, niche expansion is more popular than niche unfilling into novel environmental conditions. Annual mean temperature, annual precipitation, and precipitation of driest month may contribute to such a shift. In addition, we identified several key climatic factors and morphological traits that tend to be associated with niche expansion in *S. boulengeri* clades correspondingly. We found phenotypic plasticity [i.e., length of lower arm and hand (LAHL), hind‐limb length (HLL), and foot length (FL)] and evolutionary changes [i.e., snout–vent length (SVL)] may together contribute to niche expansion toward adapting novel niche, which provides us a potential pattern of how a colonizing toad might seed a novel habitat to begin the process of speciation and finally adaptive radiation. For these reasons, persistent phylogeographic divisions and accompanying divergences in niche occupancy and morphological adaption suggest that for future studies, distinct genetic structure and morphological changes corresponding to each genetic clade should be included in modeling niche evolution dynamics, but not just constructed at the species level.

## Graphical abstract

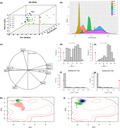

## 1 INTRODUCTION

A major goal of ecology is in the inspection of niche evolution dynamics to explain rapid lineage diversification and mechanisms of morphological evolution across clades, especially in complex mountainous regions. As reviewed by Smith et al. (2018), most niche models have been constructed at the species level to model a species responding to the environment as a single undifferentiated entity. For example, several studies have found evidence for climatic‐niche conservatism among species (Crisp et al., 2009, Kozak & Wiens, 2006, Kozak and Wiens, 2010, Liu et al., 2020a), while others have shown evidence for niche divergence (Evans et al., 2009; Graham et al., 2004; Hu et al., 2015; Knouft et al., 2006). Either way, these practices ignore whether occurrence data represent a single evolutionary entity or a collection of evolutionary lineages that can vary in age, evolutionary independence, and genetic distinctiveness (Pearman et al., 2010). Moreover, the potential effects and implicit meanings of intraspecific niche evolution dynamics across clades within species level are seldom known (Tingley et al., 2016).

In fact, niches of species or clades clearly do evolve, and niche shifts in range limits as a result of such evolution (Peterson & Holt, 2003). Both ecological (available empty niches) and evolutionary changes (genetic drift or through selection) can potentially allow a species or clade to shift into a novel niche, and an observed shift can equally result from a change of the realized niche and the fundamental niche (Broennimann et al., 2007). Realized niche shifts between native and non‐native populations can be accurately evaluated by niche expansion (i.e., species colonizing novel environmental conditions in their non‐native range Petitpierre et al., 2012; Tingley et al., 2016) and niche unfilling (i.e., partial filling of the native niche in the native range, Petitpierre et al., 2012). The fundamental niche depicts the ecophysiological requirements of species, which can be viewed as the envelope of environmental (abiotic) conditions allowing populations to sustain themselves in an n‐dimensional environmental space (Guisan et al., 2014; Soberón, 2007). In addition, over some temporal and spatial scales, intraspecific niche evolution and ecological innovation have taken place, such as in Mexican birds (Peterson & Holt, 2003). A growing number of cases indicate the evolutionary shifts occurred in range limits with rapidly changing environments (Davis & Shaw, 2001; Evans et al., 2009; Peterson & Holt, 2003; Thomas et al., 2001). Moreover, researchers have documented morphological evolution is strongly influenced by ecological niche shifts in passerine birds (Alström et al., 2015), chestnut‐capped brushfinches (Moreno‐Contreras et al., 2020), Eurasian perches (such as *Perca fluviatilis*, Bartels et al., 2012), and bivalved scallops (Sherratt et al., 2017).

Recently, combining molecular information and niche evolution models to analyze niche shifts has provided new insights into the roles of abiotic climate and geographical conditions in shaping range limits. There are three main reasons to incorporate evolutionary processes into niche modeling to assess niche and morphological evolution dynamics across clades within *Scutiger boulengeri* under climate change. First, the existence of cryptic species and frequent local adaptation suggest that cryptic niche architecture exists within the species‐level taxa that are the focus of studies of clade evolution process, ecological niche, and biotic responses to climate change (Pearman et al., 2010). Second, persistent morphological divergence is caused by genetic drift or through selection under local adaptation to environmental heterogeneity. Third, niche models, pooled from the entire range of the species, assume that species respond to the environment as an undifferentiated entity along their entire distribution but underestimating differences between distinct niches caused by range limits and local adaption (Peterson et al., 2011). In fact, spatial heterogeneity in environments coupled with reduced gene flow (resulted from intraspecific competition or dispersal limitations) can encourage local adaptation, leading to divergence in niches among closely related lineages (Smith et al., 2018). However, numerous researchers constructed niche models just by pooling but ignoring intraspecific lineages for widely distributed species owning phylogeographical structures may lose sight of considerable variation in morphological, physiological, and life‐history traits under niche evolution dynamics across clades within species (Barria et al., 2020).

The Qinghai–Tibetan Plateau (QTP)—the largest continental highland on Earth—is a major barrier to airflow in the atmosphere, which triggers the onset of the Indian summer monsoon (Molnar et al., 1993). Tibet continuously grew northward over millions of years in response to the thickening of Earth's crust associated with the collision of the Indian and Asian continental plates (Harrison et al., 1992), which is a long‐standing topographic feature that arose from the collision between India and Asia (Rowley & Currie, 2006). The orogeny of high mountain ranges separating deep valleys might have created geographical barriers reducing gene flow between isolated populations and promoted allopatric divergence (Favre et al., 2015). Meanwhile, novel environmental spaces released from biotic and abiotic constraints (Callaway & Maron, 2006; Hierro et al., 2005) would have provided key opportunities for occupation of novel niches especially in the early stages of clade divergence.

*Scutiger boulengeri*, an endemic Tibetan toad occurring in mountain streams from the South‐Tibetan (Hofmann et al., 2017), has a wide range of distributions along the eastern and southern slopes of the QTP at elevations between 2,400 and 5,270 meters above sea level (Chen et al., 2009; Subba et al., 2015). Several geographically structured haplotypes have also been identified using mitochondrial DNA and grouped into 3 major clades due to incomplete sampling (Li et al., 2009). Intraspecific clade diversity of *S. boulengeri* implies each clade resulted from unique patterns of limited migration, isolation, and local adaptation (Potter et al., 2013) across drainages along the margins of QTP. Such a promising case presents us an attractive system to study the link between intraspecific niche evolution and phenotypic evolution dynamics. The capacity for rapid phenotypic evolution may directly facilitate species diversification by increasing the ability of a radiating clade to exploit ecological opportunities (Parent & Crespi, 2009). Moreover, it also provides an ideal model to study phenotypic plasticity, which as the main means to cope with changing ambient conditions on a shorter time‐scale in ectotherms (Angilletta, 2009; Seebacher & Franklin, 2011).

Given unique intraspecific lineages, the potential for distinctive climate niches and the need to model niche evolution dynamics of *S. boulengeri* at the intraspecific clade level have been recognized. Herein, based on a set of climatic and morphological data, we applied multiple robust models and methods for incorporating evolutionary processes into niche modeling to assess niche and morphological dynamics across clades within *S. boulengeri*. Specifically, we focus to address four key issues: (a) Is there niche divergence caused by niche shifts across clades? (b) Is such a divergence caused by niche unfilling or niche expansion? (c) Which climate variables contribute most to such niche evolution dynamics? (d) Is there related trait evolution accompanied by a shifted niche when controlling for phylogenetic relatedness? We hypothesize that genetically isolated *S. boulengeri* clades would exhibit clearly segregated niche patterns and corresponding morphological variations in this system.

## 2 MATERIAL AND METHODS

### 2.1 Phylogenetic analysis

Based on previous studies (Hofmann et al., 2017; Li et al., 2009) and our own field works in recent years, we compiled 2 published mtDNA cytochrome b (cytb) genomes (GenBank IDs FJ463132 and EU180928) plus 5 newly obtained cytb genomes (GenBank IDs MW600725–MW600729). To construct a phylogeny for *S. boulengeri* clades, we used MEGA‐X (Kumar et al., 2018) to align six selected mtDNA cytb genomes with one genome from outgroup *Oreolalax omeimontis*. Phylogenetic trees were constructed separately by using maximum likelihood (ML) and Bayesian inference (BI) methods, both of which were implemented in PhyloSuite v1.2.1 (Zhang et al., 2020). The best‐fit BIC substitution model (TPM2 + F + G4) was selected in ModelFinder (Kalyaanamoorthy et al., 2017). Divergence time for the reconstructed trees was estimated with the RelTime ML method using MEGA‐X (Kumar et al., 2018). Based on previous researches, we choose the most recent common ancestor (MRCA) of *Scutiger* and *Oreolalax* (53 Ma) as the calibration point (Hofmann et al., 2017).

### 2.2 Occurrence and environmental data

**FIGURE 1:**
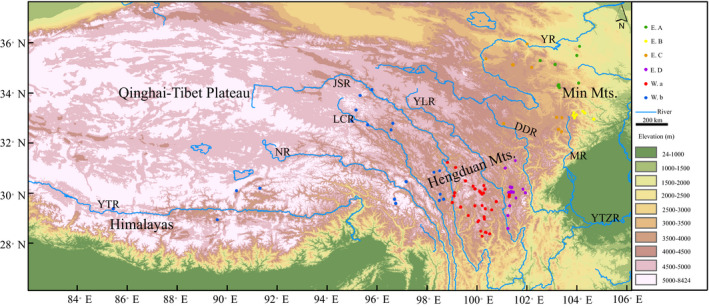
Clades’ distributions based on occurrence records for *Scutiger boulengeri*. Map showing the Tibet regions including main mountains and main rivers. Abbreviations: YTR, Yarlung Tsangpo River; NR, Nu River; LCR, Lancang River; JSR, Jinsha River; YLR, Yalong River; DDR, Dadu River; MR, Min River; YTZR, Yangtze River; YR, Yellow River; Mts., Mountains

We obtained occurrence records for *Scutiger boulengeri* from our own field works and the published literatures (Hofmann et al., 2017; Li et al., 2009). Localities cover Himalayas, QTP, Hengduan Mountains, Min Mountains, and adjacent mountains (Figure 1).

We compiled 19 BIOCLIM variables and elevation for each period from the WorldClim database with a resolution of 30 s (~1 km) for each environment layer (Fick & Hijmans, 2017). We included the dissimilarity of the enhanced vegetation index variable, drawn from the Global Habitat Heterogeneity project (Tuanmu & Jetz, 2015). Because strong colinearity between environmental variables could inflate model accuracy (Boria et al., 2014; Veloz, 2009), we examined pairwise correlations among the 21 variables within each clade distribution. We reduced autocorrelation of input environmental data by removing highly correlated variables with the threshold of Pearson's correlation tests |*r*| > .8 (Dormann et al., 2013). Finally, we chose 8 variables with lower correlation for subsequent analyses.

### 2.3 Model evaluation of ecological niche models (ENMs)

We used the ENMeval R package to facilitate increased strictness in the development of Maxent models (Muscarella et al., 2014). The Akaike information criterion corrected (AICc) for small sample sizes reflects both model goodness‐of‐fit and complexity. The model with the lowest △AICc value (i.e., △AICc = AICc − AIC_min_ = 0) is considered as the best model out of the current suite of models. We explored models with regularization multiplier (RM) values ranging from 0.5 to 4.0 (increments of 0.5) and with six different feature classes (FCs) combinations as suggested by Muscarella et al. (2014).

### 2.4 Suitable habitat prediction

We used a maximum entropy modeling algorithm implemented in the program Maxent v.3.4.1 (Phillips et al., 2006; Phillips & Dudík, 2008) to predict suitable habitat. Maxent uses environmental variables from localities at which a species has been documented previously to build a predictive model of where else the clades may occur due to the presence of similar environmental conditions (Elith et al., 2011). To assess model performance, we calculated the average value of the area under the receiver operating characteristic curve (AUC) for training and testing datasets (Swets, 1988), AUC takes on values ranging from 0.5 (no better discrimination than random) to 1 (perfect discrimination).

### 2.5 Niche overlap and null hypothesis test

One key assumption for applying ENMs is that species’ niche changes very slowly across space and time (Warren et al., 2008). These tests are based on two similarity metrics (Warren's I and Schoener's D); we calculated these metrics in ENMTools v1.4.3, using 100 replicates to generate a pseudoreplicated null distribution (Warren et al., 2010). The null hypothesis of niche equivalency is rejected when empirical values are significantly less than the critical values for both the niche equivalency and similarity tests (Warren et al., 2010).

Climate niche overlap in E‐space between lineages of *S. boulengeri* was estimated using the PCA‐env approach proposed by Broennimann et al. (2012). An unbiased estimate of the Schoener's D metric can be calculated for our data and is ensured to be independent of the resolution of the grid; statistical confidence in niche overlaps was then tested through a bidirections niche similarity test (Broennimann et al., 2012).

### 2.6 Niche expansion or unfilling

Schoener's D on species occupancy disentangles climate availability and the extent of niche divergence of clade pairs, but does not take into account the difference between partial filling and expansion (Petitpierre et al., 2012). However, expansions measured can characterize true niche shifts, when native and non‐native ranges overlapped in climatic space, Following Petitpierre et al. (2012), three categories were considered in: (a) stable environments where species occurs in both ranges, (b) unfilled environments where species occur only in the native range, and (c) expansion environments where the species occur only in the non‐native range.

### 2.7 Niche evolution

We used phytools R package (Revell, 2013) to visualize niche evolution throughout the phylogeny. We calculated the K value for a given trait and phylogeny; phytools package provides a randomization test to assess the significance of the observed K value (Revell, 2020). Finally, to assess the evolution mode along each climatic‐niche component (Cooper et al., 2010), we fit four alternative models of evolution for values of each climatic principal components (PCs): (a) Brownian motion model (BM) (Felsenstein, 1985); (b) “Single‐peak” (OU) (Butler & King, 2004; Hansen, 1997); (c) an early‐burst model (EB) (Harmon et al., 2010); and (d) a white noise model (WN). Calculations were conducted using the geiger package (Harmon et al., 2007), and the best‐fitting model was chosen using the Akaike information criterion corrected (AICc) for small sample sizes and Akaike weights (ω) (Wagenmakers & Farrell., 2004).

### 2.8 Morphological data

These data have been derived from 151 specimens from our recent field works and Herpetological Museum of the Chengdu Institute of Biology, CAS. The sample sizes varied between 8 and 42 specimens per clade (only male adults included), with a mean of 25 individuals per clade. The morphological variables include the following: snout–vent length (SVL), head length (HL), head width (HW), snout length (SL), internasal space (INS), width of upper eyelid (UEW), interorbital space (IOS), diameter of eye (ED), length of lower arm and hand (LAHL), diameter of lower arm (LAD), hind‐limb length (HLL), tibia length (TL), tibia width (TW), length of foot and tarsus (TFL), and foot length (FL). In addition, for other named system of morphological variables, we follow Fei et al. (2005). *Scutiger boulengeri* is characterized by one or two pairs of keratinized spine patches on the chests of males, a reduced columella, and the absence of a tympanum (Chen et al., 2009). In data analysis, we removed females for their insufficient quantity. Prior to all statistical analyses, the variables were log‐transformed to better meet the requirements of normality and homogeneity (Rabosky & Adams, 2012).

### 2.9 Phylogenetic comparative methods (PCMs) and trait correlative analyses

Biologists have long recognized that closely related species are generally more similar to one another than they are to more distantly related, which is often termed phylogenetic conservatism (Martins & Hansen, 1997). Phylogenetic signals can be considered as the degree to which similarity in trait values between species can be predicted upon their relatedness (Harvey & Rambaut, 2000). If there are phylogenetic signals in the data, then PCMs are necessary for robust statistical analyses of trait correlations. To address the relationships between morphology and climate variables caused by niche evolution, we used phylogenetic generalized least squares (PGLS, Grafen, 1989; Martins & Hansen, 1997). The workflow, incorporating all the methods and processes for modeling climatic‐niche evolution dynamics and key morphological changes along phylogenetic clades in *S. boulengeri*, is presented in the Supplementary material Appendix Figure A1.

## 3 RESULTS

### 3.1 Phylogeny

**FIGURE 2:**
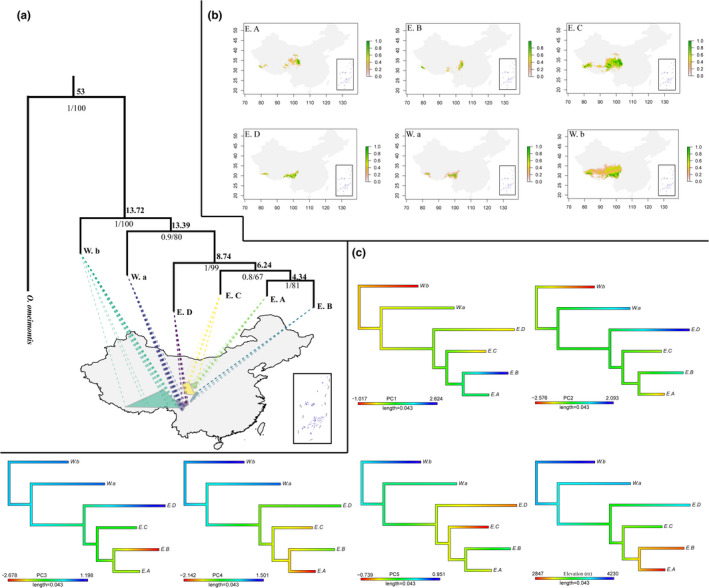
(a) Phylogenetic relationships and geographic distributions in minimum convex polygon (MCP) and the suitable range of six clades in *Scutiger boulengeri*; supporting values of BI and ML labeled on branches; divergence time (Ma) of dated tree in bold; (b) the suitable habitat range prediction map for each clade; (c) ancestral state reconstructions of main climatic PCs and elevation, colors of branches reflect values of PCs, and elevation interpolating the states along each edge

We identified *Scutiger boulengeri* containing six clades: E. A, E. B, E. C, E. D, W. a, and W. b. Due to the consensus trees of the maximum likelihood (ML) and Bayesian inference (BI), we present only the ML tree for mtDNA genes of the *S. boulengeri* clades in Figure 2a. The tree has high reconstruction confidence as the supporting values of the internal nodes are very high.

Molecular dating supports the earliest clade divergence of W. b (from Himalayas and QTP) to W. a (from the Hengduan Mountains) during the Miocene (13.72 Ma). Nearly at the same period around 13.39 Ma, W. a clade split from E. D, but the remaining clades gradually diverged around ca. 4–8 Ma.

### 3.2 Occurrence and environmental data

Our final dataset comprises 96 georeferenced occurrence records: 42 for east represented by 4 clades (9 for E. A clade, 9 for E. B, 10 for E. C, 14 for E. D) and 54 for west represented by 2 clades (35 for W. a clade and 19 for W. b).

**FIGURE 3:**
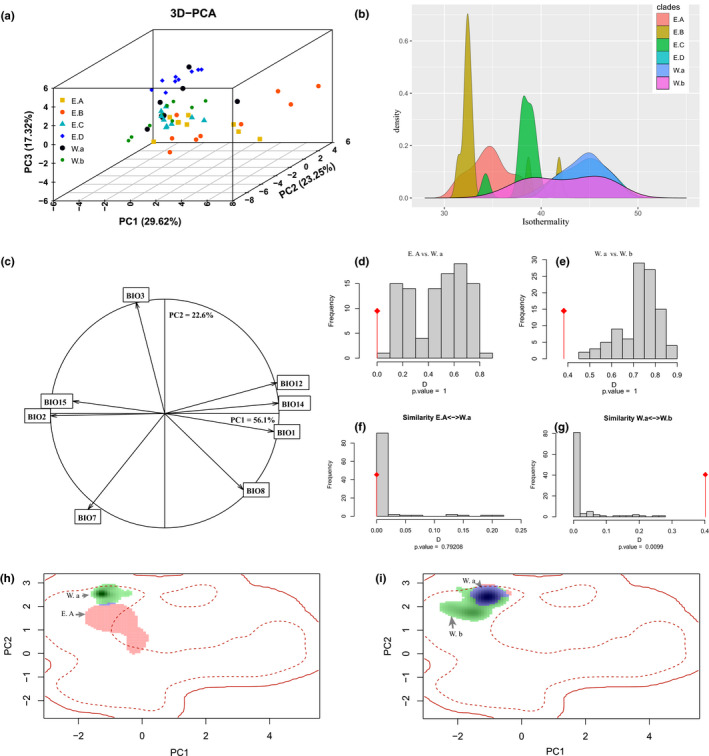
(a) Three‐dimensional principal component analysis (3D‐PCA) of climate variables across six clades within *Scutiger boulengeri*; (b) predicted occupied niche of isothermality across six clades within *S. boulengeri*; (c) correlation and contribution of each variable to the first two components of the PCA‐env; (d‐e) histograms of niche equivalency distributions, diamond lines represent observed values; (f‐g) histograms of niche similarity distributions in bidirections; (h‐i) two pairwise comparisons of niche dynamic between native and shifted ranges in environmental space depicted by the first two axes of a PCA, calibrated on the entire range of conditions available in China (red solid lines). Niche expansion, overlap, and unfilling situations are stacked in the environmental space for each clade. Green areas represent climates only occupied in the native range, and blue areas indicate climates occupied in both the native and non‐native range, while red areas indicate niche expansion in the shifted range. Shading indicates the density of occurrences of the species by cells in the native range. The solid and dashed contour lines illustrate, respectively, 100% and 50% of the available environment in the native range

According to our principal component analysis (PCA) of 21 environmental variables, the top five principal components explain 91.14% of variations (Table 1). PC1 is mainly represented by the increased temperature and reduced elevation, while PC2 explains increased precipitation, PC3 explains mean diurnal range and precipitation seasonality, PC4 and PC5 both represent Min temperature of coldest month and temperature annual range. The top three principal components are presented in Figure 3a; for more details, please see Table 1.

**TABLE 1:** Loadings on the top five principal components

		PC1	PC2	PC3	PC4	PC5
BIO1	Annual mean temperature	**0.935**	0.271	0.037	0.219	−0.033
BIO2	Mean diurnal range	−0.187	−0.173	**0.819**	0.153	−0.199
BIO3	Isothermality	−0.240	0.320	**0.686**	0.518	−0.205
BIO4	Temperature seasonality	0.227	−0.707	−0.182	−0.550	0.171
BIO5	Max temperature of warmest month	**0.983**	−0.083	0.000	0.028	−0.004
BIO6	Min temperature of coldest month	0.034	−0.396	0.217	**0.655**	**0.563**
BIO7	Temperature annual range	0.121	0.383	−0.218	**−0.651**	**−0.565**
BIO8	Mean temperature of wettest quarter	0.905	0.230	0.055	−0.119	0.269
BIO9	Mean temperature of driest quarter	0.703	0.243	0.039	0.586	−0.264
BIO10	Mean temperature of warmest quarter	**0.994**	0.046	−0.035	0.050	0.018
BIO11	Mean temperature of coldest quarter	0.783	0.465	0.088	0.383	−0.081
BIO12	Annual precipitation	−0.173	0.872	−0.348	−0.137	0.051
BIO13	Precipitation of wettest month	−0.176	**0.939**	0.191	−0.033	0.135
BIO14	Precipitation of driest month	−0.361	0.241	−0.624	0.353	0.347
BIO15	Precipitation seasonality	−0.012	−0.080	**0.879**	−0.176	0.266
BIO16	Precipitation of wettest quarter	−0.213	**0.935**	0.127	−0.124	0.141
BIO17	Precipitation of driest quarter	−0.317	0.206	−0.770	0.431	0.097
BIO18	Precipitation of warmest quarter	−0.216	**0.917**	0.153	−0.166	0.217
BIO19	Precipitation of coldest quarter	−0.224	−0.231	−0.500	**0.634**	**−0.425**
	Dissimilarity	0.199	−0.123	−0.278	−0.142	0.383
	Elevation	−0.914	−0.055	0.274	0.232	−0.048
	Eigenvalue	6.221	4.883	3.638	2.882	1.516
	Percentage of variance (%)	29.623	23.253	17.322	13.723	7.217

Note

We retained all principal components with eigenvalues greater than 1. Loadings occupied the top three are given in bold for each component. PC1 mainly corresponds to temperatures, while PC2 mainly corresponds to precipitation.

### 3.3 Prediction of the Maxent distribution

We chose the best model and found the corresponding RM and FC parameters for Maxent model to facilitate increased rigor in the development of Maxent models (Table 2). The distributions based on Maxent across *S. boulengeri* clades are characterized by high AUC statistics, indicating that these ENMs successfully discriminate real occurrences from background locations. The jackknife tests on variable importance for *S. boulengeri* clades reveal that precipitation of driest month in E. A, E. B, and W. a clades, while annual precipitation in E. C and E. D and annual mean temperature in W. b produce the greatest decrease in gain when excluded from the model, suggesting these climate factors limit distributions of clades correspondingly, which are likely the most important reasons for the next step niche divergence.

**TABLE 2:** The parameters and relative contributions of the environmental variables in Maxent model

Clade	Parameter	AUC	BIO1	BIO2	BIO3	BIO7	BIO8	BIO12	BIO14	BIO15
E. A	LQ1	0.979	**0.231**	0.033	0.12	0	0	0.163	0.301*	0.153
E. B	LQ1	0.977	0.012	0.313	0	0.026	0	**0.097**	0.308*	0.172
E. C	LQ2	0.937	0.194	0	**0.368**	0.001	0.006	0.255*	0.174	0.001
E. D	LQ3	0.99	0.244	0	**0.373**	0.01	0	0.254*	0.119	0
W. a	L2.5	0.892	0.062	0	**0.703**	0.029	0.026	0	0.174*	0.002
W. b	LQH4	0.914	0.652*	0	0.184	0	0	0.115	0.049	0

Note

The environmental variables with the highest gain when used in isolation are given in bold for each clade; the variables that decrease the gain the most when they are omitted are given * for each clade.

Abbreviations: H, hinge; L, linear; Q, quadratic.

### 3.4 Hypothesis tests based on ENMs and PCA‐env approaches

ENM‐based niche equivalency tests reveal that four paired comparisons do not reject null distributions, seem equivalent as the values of observed niche overlap fall well in the middle of the null distributions (Supplementary material Appendix Figure A2). ENM‐based background similarity tests indicate greater niche divergence between pairwise comparisons (2/15): E. B versus W. b and E. A versus E. B (Supplementary material Appendix Figure A3a,i). Interestingly, some pairwise comparisons (7/15) just show one‐sided significant divergence. The last part (6/15) niche overlap falls within the 95% confidence limits of the null distributions, leading to nonrejection of the hypothesis of retained niche conservatism.

The results of PCA‐env‐based niche equivalency test (Figure 3d,e and Supplementary material Appendix Figure A4) show that pairwise comparisons of 80% (12/15) less than expected null distribution ranges and reject null hypotheses, indicating closely related clades are not equivalent to most related clades, and most clades have undergone significant alterations of their environmental niche, and clades may be more resilient to climate changes than their native ranges suggest. Niche background test indicates that 11 paired comparisons show a very limited niche overlapping values (scores < 0.3). Only four paired comparisons show niche overlap categorized as a moderate overlap (0.307–0.527). On the other hand, the background similarity tests indicate generally little niche similarity among the pairwise comparisons in six clades (Table 3 and Supplementary material Appendix Figure A5). The ordination null tests of niche similarity show that niches are less similar than random expectations in 30% (five paired comparison cases) in reciprocal directions, while 26% (four paired comparisons) are significantly not to reject the null hypothesis of niche conservatism in bidirections.

**TABLE 3:** Niche changes indices between native range and non‐native clades

Clades	Native clades	D	*p*‐value	E	S	U
E. A	E. B	0.252	.881	**0.610**	0.390	0.603
E. A	E. C	0.220	.980	0.485	**0.515**	0.302
E. B	E. C	0.110	.940	**0.700**	0.300	0.611
E. A	E. D	0.001	.743	**0.997**	0.003	0.986
E. B	E. D	0.011	.812	**0.956**	0.044	0.744
E. C	E. D	0.095	.842	**0.907**	0.093	0.656
E. A	W. a	0.002	.792	**0.990**	0.010	0.979
E. B	W. a	0.002	.782	**0.986**	0.014	0.970
E. C	W. a	0.065	.782	**0.894**	0.106	0.838
E. D	W. a	0.459	.**010**	0.159	**0.841**	0.289
E. A	W. b	0.070	.822	0.715	0.285	**0.752**
E. B	W. b	0.017	.683	**0.913**	0.087	0.899
E. C	W. b	0.307	.**050**	0.304	**0.696**	0.493
E. D	W. b	0.444	.**020**	0.037	**0.963**	0.628
W. a	W. b	0.527	.**010**	0.031	**0.969**	0.514

Note

Boldface values indicate significant similarity with *p* < .05 and the preferred niche shift model.

Abbreviations: D, Schoener's D: the overlap value between pairwise clades; E, niche expansion; *p*‐value, the *p*‐value of the niche similarity test; S, stability; U, unfilling.

### 3.5 Niche expansion and unfilling dynamics

As predicted, examining patterns of niche expansion and niche unfilling demonstrate a gradient of realized niche change across clades in their shifted ranges (Supplementary material Appendix Figure A6). There is considerable evidence of expansion when comparing the realized niche of clade to its native niche, such as E. A clade shows 99.7% niche expansion (vs. native clade E. D) (Figure 3h). From our results (Table 3), there is nearly 50% (3/6; clade E. A, E. B and E. C) of the clades’ non‐native niche exists in climates that are less occupied in its native range (i.e., niche expansion). While 33% (2/6; clade E. D and W. a) of the clades’ native niches remain stable, only one clade W. b can be viewed as typical niche unfilling (Figure 3i).

### 3.6 Niche evolution

The results of phylogenetic signal tests based on Blomberg's K show values in PC1 and PC2 are less than 1, best‐fitted to WN model, which suggests that PC1 and PC2 dimensional climate changes are independent of phylogenetic relationships (data with no covariance structure among clades). But the values of more than 1 in PC3, PC4, and PC5 indicate PC3, PC4, and PC5 dimensional changes have closely related phylogenetic relationships (Table 4). The comparisons of model fit based on the AICc values and AICc weights (**ω**) indicate that the WN model is preferred in PC1 and PC2, where climatic components change in PC1 and PC2 regardless of shared ancestry between clades. Although niche conservatism is maintained in some clades for PC1 and PC2 (Figure 2c and Table 4), our tests of PC3, PC4, and PC5 fitted to a BM model, suggesting that following a divergence event along these climate PCs, clades branches may be subject to these variable environmental conditions, such as promoting the evolution of different climatic tolerances, which may be accumulated independently from ancestral ones.

**TABLE 4:** Phylogenetic signal and model fitting analyses obtained for five climate principal components and elevation

	K (*p* ‐ value)	BM			OU			EB			WN		
		−lnL	AICc	ω	−lnL	AICc	ω	−lnL	AICc	ω	−lnL	AICc	ω
PC1	0.950 (.21)	−9.744	27.488	0.428	−9.719	37.438	0.003	−9.744	37.488	0.003	**−9.465**	**26.930**	**0.566**
PC2	0.749 (.33)	−12.033	32.066	0.353	−12.009	42.017	0.002	−12.033	42.066	0.002	**−11.434**	**30.868**	**0.642**
PC3	1.141 (.09)	**−9.781**	**27.561**	**0.627**	−9.780	37.559	0.004	−9.781	37.561	0.004	−10.324	28.648	0.364
PC4	1.648 (.04)	**−9.266**	**26.532**	**0.665**	−8.941	35.881	0.006	−9.262	36.523	0.005	−9.986	27.972	0.324
PC5	1.381 (.049)	**−4.858**	**17.716**	**0.572**	−4.858	27.716	0.004	−4.847	27.693	0.004	−5.166	18.332	0.420
Elev.	1.568 (.045)	**−45.06**	**98.121**	**0.687**	−45.06	108.121	0.005	−45.06	108.121	0.005	−45.877	99.754	0.304

Note

Boldface values indicate the preferred model.

Abbreviations: AICc, corrected Akaike information criterion; BM, Brownian motion; EB, Early burst; Elev., Elevation; K, Blomberg's K; −ln L, values for Neperian logarithm; OU, Ornstein–Uhlenbeck with one adaptive peak;WN, White noise; ω, Akaike weights.

### 3.7 Morphological evolution

Blomberg's K‐based test of phylogenetic signal analysis shows high values (K > 1) in morphological traits in SVL (snout–vent length), SL (snout length), and INS (internasal space) indicating relatively more phylogenetic signals. The comparisons of model fit based on the AICc and AICc weights (ω) indicate the BM model is preferred in SVL, SL, and INS, where trait changes between clades can be predicted upon their relatedness. Following a divergence event along phylogeny, that is less labile than expected under a BM model of evolution, clades branches may be subject to phylogeny, such as promoting the evolution of different morphological traits under climatic tolerances, which may be accumulated independently from ancestral ones, while the others fitted a WN model, which seems to be unrelated with shared ancestry but preference of an adaptively phenotypic plasticity (Table 5), which may be closely related to habitat types adapted by distinct genetic clades.

**TABLE 5:** The results of phylogenetic signal test and model fitting analyses based on size‐corrected characteristic data

	K (*p*‐value)	BM			OU			EB			WN		
		−lnL	AICc	ω	−lnL	AICc	ω	−lnL	AICc	ω	−lnL	AICc	ω
SVL	1.222 (.07)	**8.880**	**−9.760**	**0.617**	8.886	0.228	0.004	8.880	0.240	0.004	8.382	−8.764	0.375
HL	1.066 (.12)	17.575	−27.149	0.443	17.648	−17.296	0.003	17.575	−17.149	0.003	**17.791**	**−27.583**	**0.551**
HW	0.831 (.18)	17.323	−26.646	0.410	17.410	−16.821	0.003	17.323	−16.646	0.003	**17.678**	**−27.357**	**0.585**
SL	1.109 (.08)	**23.918**	**−39.837**	**0.507**	23.971	−29.943	0.004	23.918	−29.837	0.003	23.876	−39.751	0.486
INS	1.142 (.09)	**24.476**	**−40.952**	**0.546**	24.515	−31.030	0.004	24.476	−30.952	0.004	24.276	−40.551	0.447
IOS	0.846 (.21)	21.957	−35.914	0.357	22.049	−26.098	0.003	21.957	−25.914	0.002	**22.536**	**−37.071**	**0.638**
UEW	0.606 (.57)	25.182	−42.365	0.314	25.274	−32.547	0.002	25.182	−32.365	0.002	**25.959**	**−43.918**	**0.682**
ED	0.749 (.37)	22.291	−36.583	0.310	22.409	−26.819	0.002	22.291	−26.583	0.002	**23.083**	**−38.165**	**0.685**
LAHL	0.804 (.35)	16.925	−25.850	0.296	17.013	−16.026	0.002	16.951	−15.901	0.002	**17.787**	**−27.574**	**0.700**
LAD	0.502 (.75)	20.182	−32.364	0.189	20.305	−22.610	0.001	20.182	−22.364	0.001	**21.634**	**−35.268**	**0.808**
HLL	0.565 (.66)	13.808	−19.616	0.297	13.899	−9.798	0.002	13.808	−9.616	0.002	**14.662**	**−21.324**	**0.698**
TL	0.359 (.96)	19.975	−31.951	0.096	20.180	−22.359	0.001	19.975	−21.951	0.001	**22.220**	**−36.439**	**0.903**
TW	0.376 (.93)	8.486	−8.973	0.092	8.697	0.605	0.001	8.486	1.027	0.001	**10.773**	**−13.545**	**0.906**
TFL	0.565 (.67)	12.982	−17.964	0.243	13.109	−8.218	0.002	12.982	−7.964	0.002	**14.115**	**−20.231**	**0.754**
FL	0.587 (.65)	16.235	−24.470	0.288	16.324	−14.648	0.002	16.238	−14.476	0.002	**17.132**	**−26.265**	**0.708**

Note

Boldface values indicate the preferred model.

Abbreviations: AICc, corrected Akaike information criterion; BM, Brownian motion; EB, Early burst; K, Blomberg's K; −ln L. values for Neperian logarithm; OU, Ornstein–Uhlenbeck with one adaptive peak; WN, White noise; ω, Akaike weights.

### 3.8 PCMs and trait correlative analyses

According to PGLS, SVL (snout–vent length) and LAD (diameter of lower arm) are strongly related with variables of PC2 (coefficients: 0.567, *p* = .002; dominated by precipitation) + PC3 (coefficients: −0.682, *p* = .003; mean diurnal range and precipitation seasonality) + PC4 and PC5 (PC4: coefficients: −3.28, *p* = .001; PC5: coefficients: 5.507, *p* = .001; both represent Min temperature of coldest month and temperature annual range), indicating SVL and LAD positively related with PC2 and PC5 but negatively related with PC3 and PC4 under phylogenetic models. In the same vein, HW (head width) and SL (snout length) are controlled by PC1+PC3+PC4+PC5 (*p* < .05). PC1+PC2+PC3+PC5 best fitted in ED (diameter of eye, *p* < .05) and TW (tibia width) controlled by PC1+PC2+PC3+PC4 (*p* < .05). Interestingly, UEW (width of upper eyelid) is significant to PC1 (*p* < .05) but selected model by AICc is PC1+PC2+PC3+PC4 (*p* > .05). For other best‐fitted models, please see Table 6.

**TABLE 6:** Model comparison based on AICc values between climate principal components and traits

Model	AICc														
	SVL	LAD	HW	SL	LAHL	TFL	FL	HL	HLL	TL	INS	IOS	UEW	ED	TW
PC1	40.54	−32.91	−23.63	14.46	−18.89	−10.57	−16.26	−23.58	−11.42	−26.39	−34.46	−32.73	−40.5^*^	−33.42	−3.61
PC2	48.42	−26.64	−17.77	13.55	−18.93	−10.25	−17.31	−17.65	−13	−29.11	−29.8	−27.23	−33.82	−28.04	−4.39
PC3	37.16	−27.12	−24.63	13.63	−17.82	−10.55	−16.8	−22.37	−12.91	−26.49	−35.03	−30.66	−37.43	−32.64	−3.83
PC4	44.26	−25.48	−20.55	12.84	−19.76	−11.25	−16.36	−23.03	−11.71	−28.66	−34.98	−30.37	−33.95	−30.87	−6.28
PC5	48.33	−26.09	−17.46	14.11	48.33	−10.73	−17.13	−18.54	−11.41	−26.85	−30.22	−27.43	−34.46	−27.97	−3.88
PC1+PC2	65.89	−4.16	1.84	43.32	10.15	19.38	12.46	6.04	16.76	0.88	−5.86	−3.13	−11.83	−5.48	25.2
PC1+PC3	66.02	−7.3	4.61	42.38	10.16	19.41	12.47	6.11	13.16	1.73	−5.52	−2.73	−10.51	−3.84	26.12
PC1+PC4	65.82	−14.08	4.38	42.67	9.83	18.72	13.5	1.01	18.28	−0.43	−9	−4.68	−11.53	−5.15	23.53
PC1+PC5	70.01	−6.75	6.36	44.08	9.02	18.97	12.8	4.39	18.57	3.02	−5.3	−3.02	−13.83	−3.47	26.01
PC2+PC3	66.98	−0.39	5.36	42.97	10.41	19.44	12.37	6.9	15.88	0.88	−5.09	−1.08	−7.58	−2.66	25.46
PC2+PC4	73.99	3.33	8.95	41.64	73.99	18.75	12.52	6.92	16.64	−3.19	−5.14	41.64	−3.96	−1.05	22.35
PC2+PC5	77.93	1.21	12.05	42.24	9.86	19.15	12.23	11.39	16.58	−3	−0.61	2.56	−4.49	1.79	24.32
PC3+PC4	64.83	1.06	4.66	42.76	9.96	18.75	12.07	4.73	17.07	1.02	−7.8	−1.8	−8.46	−3.44	23.5
PC3+PC5	66.64	1.69	5.37	43.3	10.39	19.01	12	6.28	17.1	3	−5.73	−0.82	−9.41	−2.65	25.83
PC4+PC5	65.24	3.28	−2.41	42.38	10.09	18.73	12.4	3.98	17.97	0.7	−12.06	−4.47	−8.94	−8.24	22.06
PC2+PC3+PC4+PC5	−103.42^*^	−159.79^*^	−143.09	−78.36	−113.52	−101.33	−108.7	−121.5	−106.91	−129.03	−139.79	−142.72	−134.88	−138.25	−117.89
PC1+PC3+PC4+PC5	−84.8	−143.73	−151.84^*^	−78.8^*^	−114.63	−101.44	−109.15	−122.99	−108.09	−127.89	−138.13	−138.56	−136.33	−140.92	−118.63
PC1+PC2+PC4+PC5	−83.85	−149.04	−141.71	−78.57	−112.79	−101.3	−108.14	−121.38	−105.5	−127.08	**−141.13**	**−147.65**	−135.4	−136.75	−111.05
PC1+PC2+PC3+PC5	−70.04	−147.83	−138	−78.14	**−116.42**	**−102.34**	**−109.78**	**−127.62**	**−109.56**	**−133.87**	−133.48	−132.34	−138.46	−161.48^*^	−126.25
PC1+PC2+PC3+PC4	−74.42	−143.58	−144.88	−78.5	−115.07	−101.8	−109.16	−124.89	−108.99	−130.89	−135.34	−134.43	**−138.52**	−149.98	−148.49^*^

Note

Boldface values indicate the preferred model.

Abbreviations: ED, diameter of eye; FL, foot length; HL, head length; HLL, hind‐limb length; HW, head width; INS, internasal space; IOS, interorbital space; LAD, diameter of lower arm; LAHL, length of lower arm and hand; SL, snout length; SVL, snout–vent length; TFL, length of foot and tarsus; TL, tibia length; TW, tibia width; UEW, width of upper eyelid.

* Significant difference with *p* < .05.

## 4 DISCUSSION

Combining molecular information and niche evolution models, our results show there are six clades contained in *S. boulengeri* by mtDNA genetic marker. Geographic structure identified from mtDNA suggests some clades resulted from unique patterns of migration, isolation, or local adaptation (Potter et al., 2013). There are three clades departed from their native niche and show niche divergence, and all the three clades shift their niches due to niche expansion but not by niche unfilling. Some climate variables may contribute to such a shift, such as annual mean temperature and annual precipitation and precipitation of driest month according to jackknife test of variable importance. We found evolutionary changes (i.e., SVL) and phenotypic plasticity (i.e., LAHL, HLL, and FL) may together contribute to niche expansion toward adapting novel niche.

Our results agree with previous progressive uplifts of Tibet (Mulch & Chamberlain, 2006). Molecular dating of clades E. D, W. a, and W. b is almost identical to the fourth uplift of high mountain ranges and aridification of Central Asia (ca. 20 to 10 Ma), and the divergence times of clades E. A, E. B, and E. C are coincident with the final extension of the last uplift (ca. 10 Ma to present) as predicted by previous studies (Favre et al., 2015). However, future researches with more detailed complementary analyses are needed to understand a link among clades molecular dating, gene flow routes, and past geological and climatic changes across these regions.

Our results show PCA‐env‐based approach well supports E. A, E. B, and partial E. C clades with significant divergence. However, ENMs‐based method just supports divergence of E. B in bidirections, while the divergence of E. A exhibits one‐sided significance, the opposite comparisons not deviated from null expectation. But inconsistent conclusions are drawn in divergence of E. C based on two methods. In literature, inconsistent conclusions regarding niche conservatism or divergence have been frequently reported based on different approaches. For example, Guo et al. (2013) applied the ordination and ENMs for the globally introduced *Phragmites australis* and found inconsistent results. Our results based on two approaches draw almost the same conclusions in one‐sided tests, but PCA‐env‐based approach is sensitive to niche divergence, while ENMs are more inclined to niche conservatism in bidirections. We analyze reasons leading to these differences further and find the native‐range model, such as ENMs, for species native to more than one area show much lower range shift, indicating a certain difficulty to predict the distribution of widespread species or clades (Liu et al., 2020b). Strubbe et al. (2013) showed that the predictive performance of native range ENMs increased with increasing niche overlap and decreased with increasing niche change. Similarly, Tingley et al. (2014) found that a native‐range ENMs under‐predicted the extent of the species’ Australian invasion. An effective strategy to improve model predictability is to develop species‐specific models or models for functional groups (Guisan & Thuiller, 2005).

In addition, we found E. A, E. B, and E. C clades within *S. boulengeri* have an obvious expansion of climatic niche (Figure 3h and Supplementary material Appendix Figure A6). Intriguingly, they shift beyond the realized niche with the new conditions but still overlap the fundamental niche, which provides positive proof that the niche conservatism hypothesis (Kozak & Wiens, 2006; Wiens, 2004) and niche divergence hypothesis (Evans et al., 2009; Graham et al., 2004) are not contradictory. We found a gradient of realized niche change in the non‐native ranges across clades within *S. boulengeri*: niche stasis in E. D (96.3%) and W. a (96.6%), niche unfilling in W. b (75.2%), and niche expansion (vs. E. D and W. a separately) in E. A (mean = 99.3%), E. B (mean = 97.1%), and E. C (mean = 90%). Our results seem to be inconsistent with the conclusion of previous studies in Petitpierre et al. (2012), in which realized niche shifts between the native and non‐native ranges were largely due to niche unfilling. Our results are also different from the results of cane toad in Tingley et al. (2014): the shift in the realized niche of the cane toad *Rhinella marina* was solely due to niche expansion. In our results, niche expansion into novel environments is more popular than niche unfilling, suggesting that our niche divergence due to niche expansion in the shifted range and thus represents true niche changes. Why did E. D and W. a fail to fill its fundamental niche in its native range? One possibility is that the presence of closely related species (*S. glandulatus* and/or *S. mammatus*) might have prevented *S. boulengeri* from colonizing suitable environments south of its present range. Indeed, previous study found there were low rates of interspecific hybridization (Chen et al., 2009). We cannot exclude dispersal limitation in the native range as a possible contributing factor, such as Jinsha River and Yalong River (Li et al., 2009), which can also enforce stable parapatric range boundaries. Future studies will be able to test this hypothesis using laboratory or field experiments.

Numerous examples of rapid adaptation in non‐native niches suggest that rapid evolution may be common during invasions in species level (Alström et al., 2015; Bartels et al., 2012; Sherratt et al., 2017). The degree to which species adapt to novel environments is important to a range of topics in ecology and evolution (Warren et al., 2008), but is of special concern for the study of intraspecific niche evolution (Tingley et al., 2016). In our study, niche divergence caused by niche expansion indeed accompanied key morphological innovations of preadaption in novel climates versus niche unfilling and stability. Our finding of significant phylogenetic signals in SVL (Table 5) and elevation (Table 4 and Figure 2c) indicates that these acquired data are not random and our results are robust. Furthermore, our findings of significant phylogenetic signals in these traits are consistent with previous studies (Blomberg et al., 2003; Freckleton et al., 2002; Oufiero et al., 2011). We found that elevation (AIC = 21.3; *p* = .002), isothermality (AIC = 24.47; *p* = .007), mean diurnal range (AIC = 29.33; *p* = .037), and Max temperature of warmest month (AIC = 29.31; *p* = .037) are significantly negative predictors of SVL under phylogenetic models, which suggest *S. boulengeri* toads from warmer and more arid environments tend to be larger, which is in concert with true records in our field works.

Several factors may underlie the observed pattern of SVL variations in *S. boulengeri* clades. One possibility pertains to the expected relationship between fasting endurance and SVL (Mautz, 1982). The second possibility is ecological release in novel shifted areas may allow for larger SVL (Losos & Queiroz, 1997; Yoder et al., 2010). The third is likely that maintenance of preferred body temperature influences the evolution of SVL (Oufiero et al., 2011). Our results highlight reduced competitors (ecological release) in a newly shifted niche may be the most likely reason for enlarged SVL in E. A, E. B, and partial of E. C clades. Furthermore, larger body sizes would likely be an advantageous trait in toads as it would enable a more generalized diet, higher fecundity, higher mobility, and greater resistance to water loss than species with smaller body sizes (Tingley et al., 2010).

Moreover, we found species tolerance of newly shifted niches tends to have morphological attributes important for locomotor performance. These traits may be a key preadaptation in toads that helps overcoming the challenge of insufficient precipitation or high temperature in novel habitats, which are in accord with the evolutionary shifts mechanistic model highlighted by prior studies (Kolbe et al., 2010; Phillips et al., 2010; Tingley et al., 2014). In our study, LAHL (length of lower arm and hand) has mean value: 27.19 mm in clade E. A, 26.27 mm in clade E. B, and 23.95 mm in E. C, and 21.77–23.57 mm in the remaining clades, and HLL (hind‐limb length) and FL (foot length) have the same trend (Supplementary material Appendix Table A1). Interestingly, these character values, without size‐correction, have a high phylogenetic signal, best‐fitted BM model. However, once size‐corrected, these character values will have a completely different scenario—with a low phylogenetic signal but trait correlations still exist, there seems to be a trade‐off strategy by locomotor performance combined enlarged SVL for speed and endurance in thermal reaction norms (Angilletta et al., 2003). Collectively, we found phenotypic plasticity (i.e., LAHL, HLL, and FL) and evolutionary changes (i.e., SVL) may together contribute to niche expansion toward adapting novel niche. Indeed, because the proximate mechanisms that underlie variations between body length and locomotor performance can be complex, quantifying the fitness consequences of the resulting trade‐offs will be challenging, novel analytical tools and optimization models are needed in further studies.

Our results show one important caveat that we were unable to conclude the accurate leading climatic variables contributing to niche expansion from niche models. In our study, we acquired the most important and limiting factors with reduced auto‐correlative variables according to the jackknife test in Maxent analysis. The results seem to be inconsistent with trait PGLS analysis. A potential explanation for our finding is that we used reduced auto‐correlative variables to increase the accuracy of Maxent model. However, in PCMs and trait correlative analyses, we used all the environmental variables to reduce data dimensions, leading to inconsistent results. These findings and potential issues provide us important inspiration and guidance for our future research.

## 5 CONCLUSION

Combining and analyzing distinct genetic clades from different geographic areas with correlative niche models and morphological evolution models, we have shown considerable variations in the degree of realized niche expansion and unfilling across the clades within *S. boulengeri* toads in the Qinghai–Tibet Plateau region. In the case of *S. boulengeri* toads, niche divergence occurs accompanied by niche expansion rather than niche unfilling, that is, niche expansion is more prevalent than niche unfilling in E. A, E. B, and E. C clades, while niche unfilling presents just in W. b clade.

Meanwhile, niche divergence caused by niche expansion indeed accompanies key morphological innovations of preadaption in novel climates than niche unfilling and stability. Factors such as enlarged body size and enhanced locomotor performance have been shown to increase expansion success by helping toads to cope with novel conditions.

Recognizing true niche shifts accompanied by key morphological innovations do exist; further assessments should seek to understand molecular mechanisms of key morphological innovations and/or related life strategies that have allowed these particular clades to expand their niches dramatically. It would be particularly interesting to use the same framework to test in the future whether the same patterns are found in other organisms, especially for other widespread species or clades.

## CONFLICT OF INTEREST

The authors declare no conflicts of interest.

## AUTHOR CONTRIBUTIONS

**Xiuqin Lin:** Conceptualization (lead); Data curation (lead); Formal analysis (lead); Investigation (lead); Methodology (equal); Software (lead); Visualization (lead); Writing‐original draft (lead); Writing‐review & editing (lead). **Chungkun Shih:** Conceptualization (equal); Formal analysis (equal); Supervision (equal); Writing‐review & editing (equal). **Yinmeng Hou:** Data curation (equal); Methodology (equal); Resources (equal); Writing‐review & editing (equal). **Xiaoxiao Shu:** Data curation (equal); Formal analysis (equal); Software (equal); Writing‐review & editing (equal). **Meihua Zhang:** Data curation (equal); Resources (equal); Writing‐review & editing (equal). **Junhua Hu:** Investigation (equal); Resources (equal); Writing‐review & editing (equal). **Jiangping JIANG:** Funding acquisition (lead); Project administration (lead); Supervision (equal); Writing‐review & editing (equal). **Feng Xie:** Conceptualization (lead); Funding acquisition (lead); Methodology (equal); Project administration (equal); Resources (lead); Supervision (lead); Writing‐original draft (equal); Writing‐review & editing (equal).

## ETHICAL APPROVAL

All animal handling and processing were in accordance with the Laws of the People's Republic of China on the Protection of Wildlife and approved by the Animal Care Committee of CIB, Chinese Academy of Sciences.

### OPEN RESEARCH BADGES

This article has earned an Open Data Badge for making publicly available the digitally‐shareable data necessary to reproduce the reported results. The data is available at https://doi.org/10.5061/dryad.2ngf1vhn6.

## Supporting information

Supplementary Material

## Data Availability

DNA sequences: GenBank accessions MW600725–MW600729. Climate data and Maxent input files: Dryad https://doi.org/10.5061/dryad.2ngf1vhn6. Sampling locations, morphological data: Dryad https://doi.org/10.5061/dryad.2ngf1vhn6.
