# Supplementary material for: Climatic‐niche evolution with key morphological innovations across clades within Scutiger boulengeri (Anura: Megophryidae)
Source: Ecol Evol. 2021 Jul 1;11(15):10353–68. doi: 10.1002/ece3.7838 (PMC8328447; doi:10.1002/ece3.7838)
Supplement: Supplementary file 1 — Supplementary Material [file ECE3-11-10353-s001.docx]

**SUPPORTING INFORMATION**

Supplementary material Appendix Table A1 Mean values of measured morphological characters.

| Group | N | SVL | HL | HW | SL | INS | IOS | UEW | ED | LAHL | LAD | HLL | TL | TW | TFL | FL |
| --- | --- | --- | --- | --- | --- | --- | --- | --- | --- | --- | --- | --- | --- | --- | --- | --- |
| E. A | 8 | 54.72 | 17.30 | 17.05 | 5.85 | 4.62 | 5.10 | 4.28 | 5.93 | 27.19 | 6.56 | 70.75 | 22.69 | 6.44 | 36.15 | 25.33 |
| E. B | 11 | 54.94 | 17.87 | 17.41 | 5.99 | 4.74 | 5.25 | 4.24 | 6.20 | 26.27 | 5.82 | 69.44 | 21.76 | 6.72 | 33.96 | 24.15 |
| E. C | 38 | 50.00 | 17.00 | 17.02 | 5.80 | 4.64 | 5.32 | 4.25 | 6.10 | 23.95 | 6.43 | 62.95 | 20.36 | 6.37 | 31.95 | 22.83 |
| E. D | 38 | 48.32 | 16.55 | 16.50 | 5.61 | 4.45 | 4.91 | 4.12 | 5.85 | 23.33 | 5.78 | 62.37 | 19.26 | 6.93 | 29.37 | 22.06 |
| W. a | 20 | 48.01 | 16.32 | 16.49 | 5.67 | 4.70 | 5.12 | 3.82 | 5.77 | 23.57 | 5.55 | 62.71 | 19.15 | 5.63 | 31.55 | 22.93 |
| W. b | 36 | 47.68 | 16.97 | 16.23 | 5.67 | 4.53 | 5.08 | 3.96 | 5.83 | 21.77 | 5.84 | 60.45 | 19.16 | 6.41 | 28.70 | 20.82 |

Note: Abbreviation: N: number of each clades.

Note: Abbreviations: SVL: snout–vent length; HL: head length; HW: head width; SL: snout length; INS: inter-nasal space; UEW: width of upper eyelid; IOS: inter-orbital space; ED: diameter of eye; LAHL: length of lower arm and hand; LAD: diameter of lower arm; HLL: hind-limb length; TL: tibia length: TW: tibia width; TFL: length of foot and tarsus; and FL: foot length.


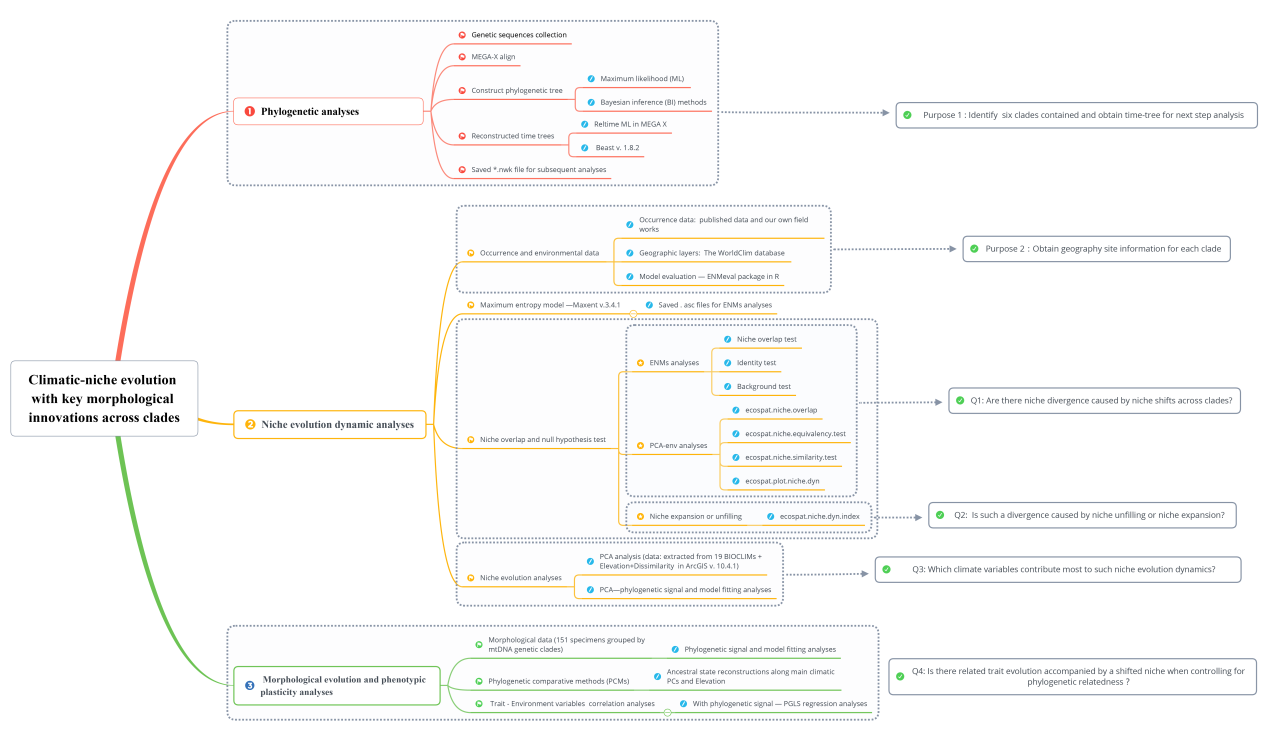
­­­

Supplementary material Appendix Figure A1. The workflow for modelling climatic-niche evolution dynamics and key morphological changes along phylogenetic clades in *S. boulengeri*.


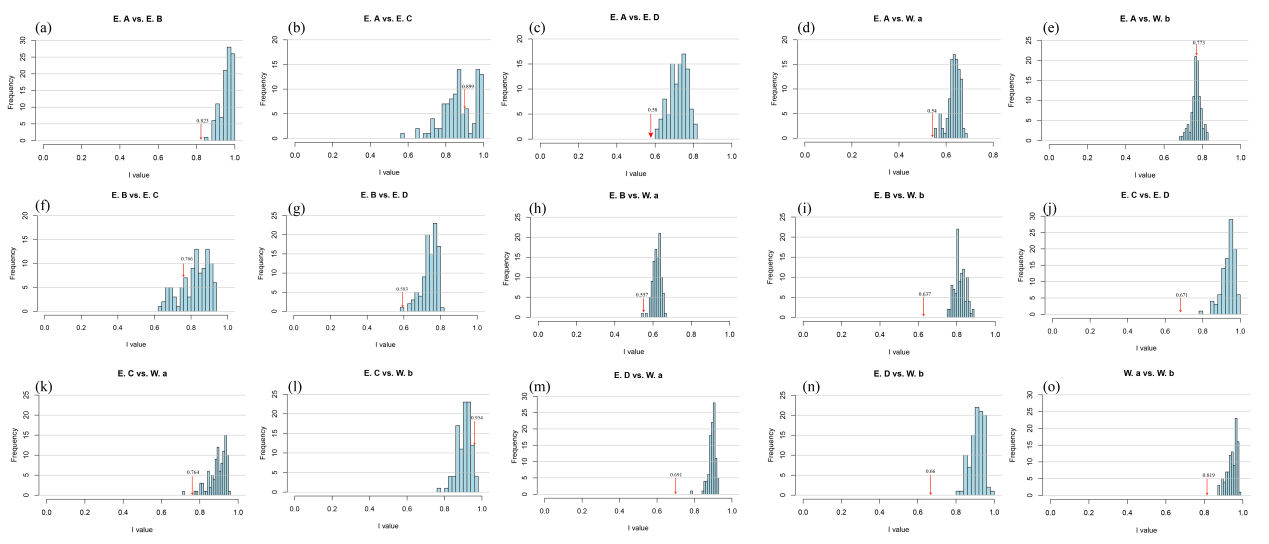


Supplementary material Appendix Figure A2. ENMs based test of niche equivalency. The histograms illustrate distributions of overlap scores from pseudoreplicates in niche equivalency tests, arrows represent observed values.


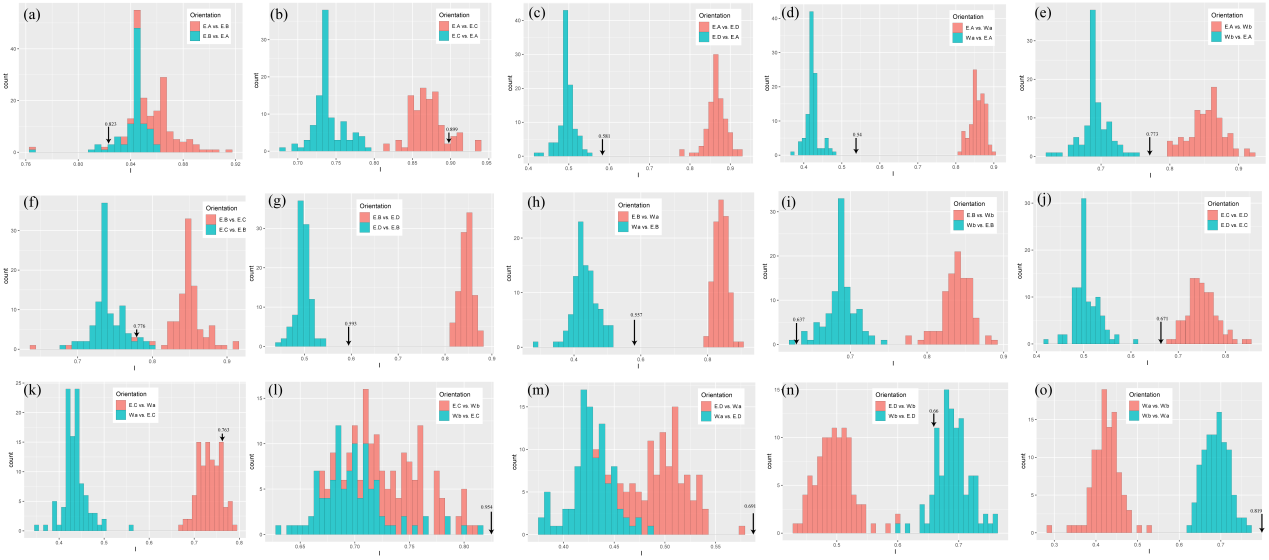


Supplementary material Appendix Figure A3. ENMs based background similarity tests. The histograms illustrate distributions of overlap scores from pseudoreplicates in niche background similarity test, arrows represent observed values.


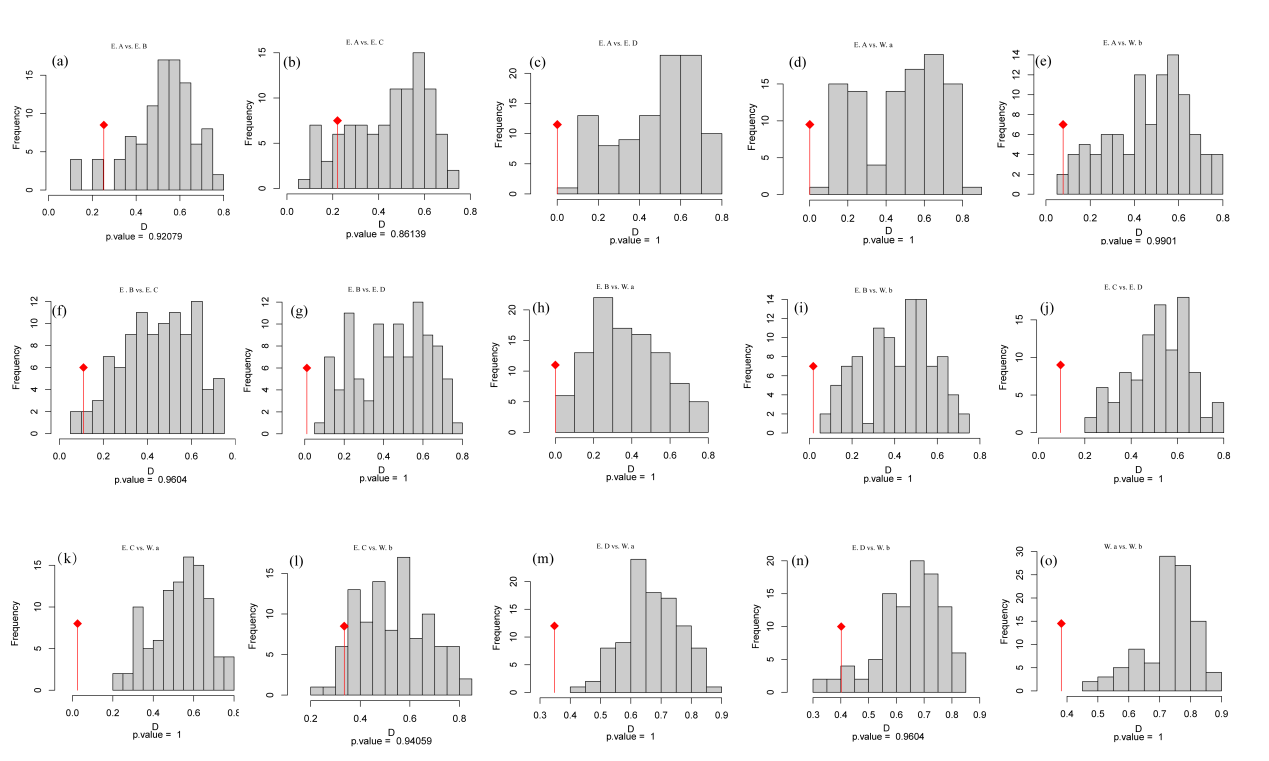


Supplementary material Appendix Figure A4. Identity tests based on PCA-env approach, pairwise comparisons between six evolutionary lineages across *Scutiger boulengeri* clades, red diamond lines represent observed values.


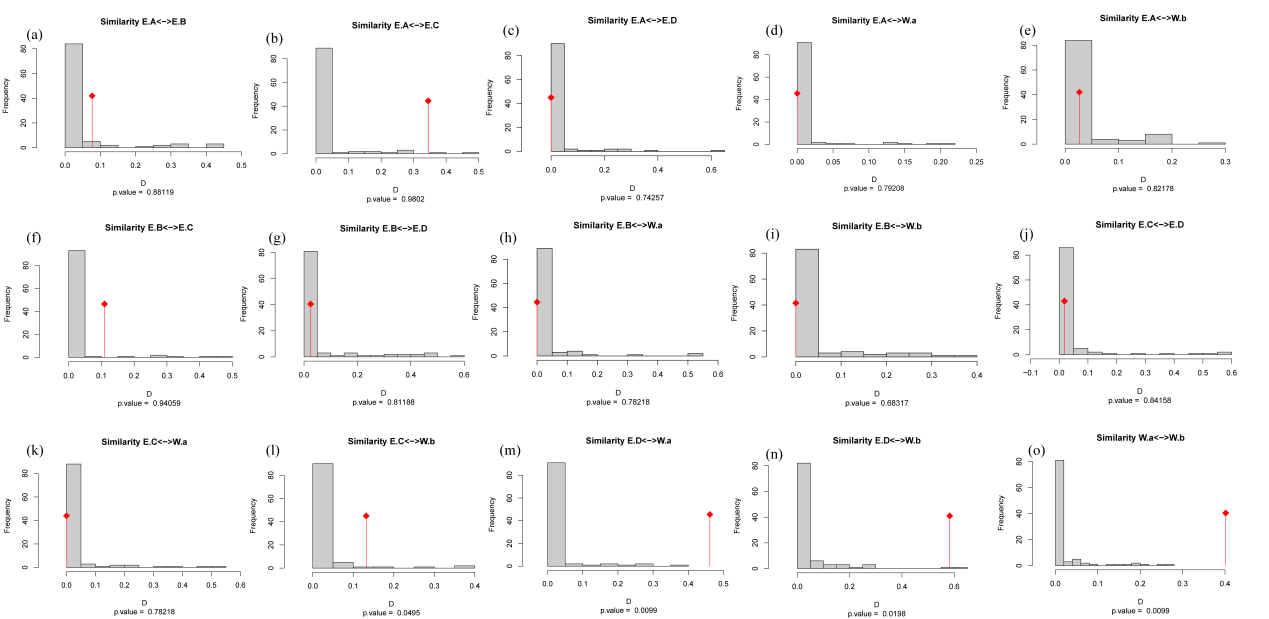


Supplementary material Appendix Figure A5. Background similarity tests based on PCA-env approach, pairwise comparisons across *Scutiger boulengeri* clades in bi-directions, red diamond lines represent observed values.


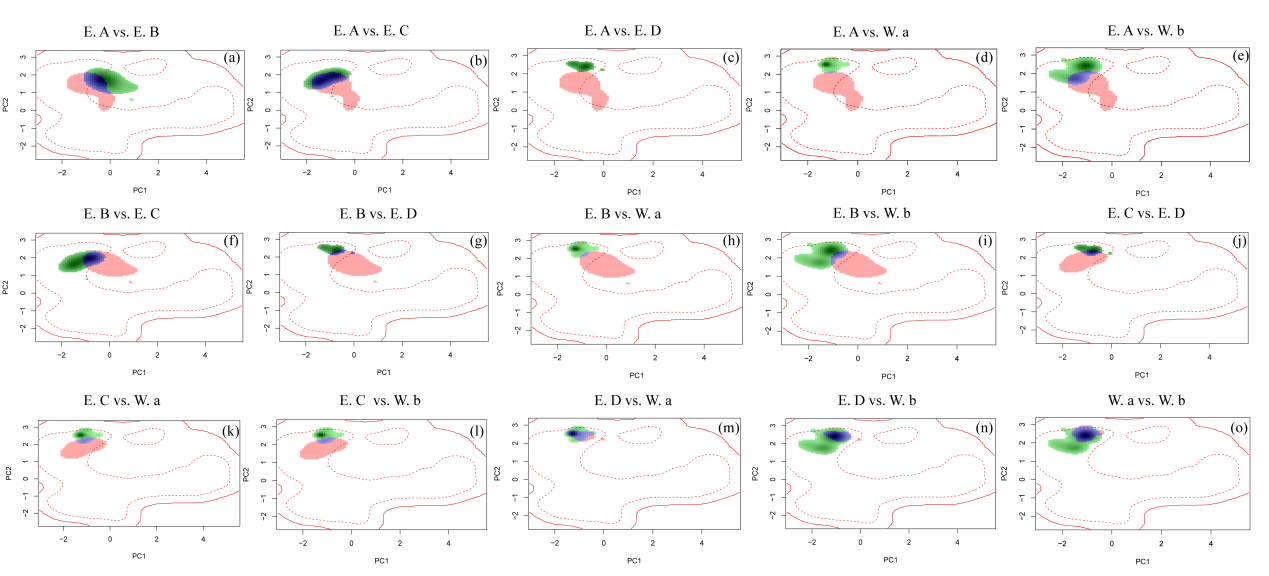


Supplementary material Appendix Figure A6. Pairwise comparisons of niche dynamic between native and shifted ranges in environmental space depicted by the first two axes of a principal component analysis, calibrated on the entire range of conditions available in China (red solid lines). Niche expansion, overlap and unfilling situations are stacked in the environmental space for each clade. Green areas represent climates only occupied in the native range and blue areas indicate climates occupied in both the native and non-native range, while red areas indicate niche expansion in the shifted range. Shading indicates the density of occurrences of the species by cell in the native range. The solid and dashed contour lines illustrate 100% and 50% of the available environment in the native range respectively.
